# Integrated Extrinsic and Intrinsic Self‐Healing of Polysiloxane Materials by Cleavable Molecular Cages Encapsulating Fluoride Ions

**DOI:** 10.1002/advs.202303655

**Published:** 2023-07-28

**Authors:** Mai Suzuki, Taiki Hayashi, Takuya Hikino, Masafumi Kishi, Takamichi Matsuno, Hiroaki Wada, Kazuyuki Kuroda, Atsushi Shimojima

**Affiliations:** ^1^ Department of Applied Chemistry Faculty of Science and Engineering Waseda University 3‐4‐1 Okubo, Shinjuku‐ku Tokyo 169‐8555 Japan; ^2^ Department of Advanced Science and Engineering Faculty of Science and Engineering Waseda University 3‐4‐1 Okubo, Shinjuku‐ku Tokyo 169‐8555 Japan; ^3^ Kagami Memorial Research Institute for Materials Science and Technology Waseda University 2‐8‐26 Nishiwaseda, Shinjuku‐ku Tokyo 169‐0051 Japan

**Keywords:** cage compound, fluoride ion, germoxane, self‐healing, silicone

## Abstract

Self‐healing ability is crucial to increasing the lifetime and reliability of materials. In this study, spatiotemporal control of the healing of a polysiloxane material is achieved using a cleavable cage compound encapsulating a fluoride ion (F^−^), which triggeres the dynamic rearrangement of the siloxane (Si–O–Si) networks. A self‐healing siloxane‐based elastomer is prepared by cross‐linking polydimethylsiloxane (PDMS) with a F^−^‐encapsulating cage‐type germoxane (Ge–O–Ge) compound. This material can self‐heal repeatedly under humid conditions. The F^−^ released by hydrolytic cleavage of the cage framework contributes to rejoining of the cut pieces by promoting the local rearrangement of the siloxane networks. The use of a molecular cage encapsulating a catalyst for dynamic bond rearrangement provides a new concept for designing self‐healing polysiloxane materials based on integrated extrinsic and intrinsic mechanisms.

## Graphical abstract

A self‐healing siloxane‐based material is created by cross‐linking linear polydimethylsiloxane (PDMS) with a cage‐type germoxane (Ge–O–Ge) compound as both cross‐linkers and molecular capsules of fluoride ions. Under humid conditions, fluoride ions are released by the cleavage of the germoxane cages and promote the rearrangement of siloxane networks to rejoin the cut surfaces.

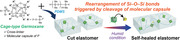

## 1 Introduction

Materials damaged by external forces require repair or replacement, which has undesirable impacts on cost, energy, and the environment. Self‐healing materials that can spontaneously repair damage have received significant interest for addressing this issue.^[^ ^1^ ^]^ Silicone elastomers consisting mainly of polydimethylsiloxane (PDMS) are widely used as coatings, sealants, and molds owing to their excellent properties, such as high thermal and chemical stability.^[^ ^2^ ^]^ However, their chemical inertness and low adhesive property make it difficult to repair cracks or rejoin cut pieces. Therefore, the design of self‐healing silicone‐based materials is of great importance.^[^ ^3^ ^]^

The introduction of organic groups that can form reversible bonds such as hydrogen bonds,^[^ ^4^ ^]^ ionic bonds,^[^ ^5^ ^]^ dynamic covalent bonds,^[^ ^6^ ^]^ and metal–ligand coordination bonds^[^ ^4^, ^7^ ^]^ into the silicone networks is a promising approach to impart intrinsic self‐healing ability. However, the Si–O–Si bonds in the main chains of these materials are not re‐formed once cleaved. Some reports have shown that the Si–O–Si bonds behave as dynamic bonds under mild conditions in the presence of nucleophiles such as silanolate (S–O^−^)^[^ ^8^ ^]^ and amine.^[^ ^9^ ^]^ This feature allows the healing of damage through the restructuring of the main Si–O–Si networks. Zheng and McCarthy reported the self‐healing behavior of cross‐linked PDMS elastomers containing reactive tetramethylammonium dimethylsilanolate end groups. These anionic end groups cleave Si–O–Si bonds via nucleophilic attack and form new Si–O–Si bonds, thus promoting the rearrangement of the networks to achieve the healing of cut pieces at 90 °C.^[^ ^8a^ ^]^ Furthermore, Schmolke et al. prepared a similar silicone gel that could heal at ambient temperature by increasing the amount of the silanolate groups in the network.^[^ ^8b^ ^]^ However, these siloxane‐based materials have major drawbacks that limit their applications. First, the Si–O–Si bonds are constantly rearranged even when no damage occurs, which causes deformation of the materials under stress. Second, equilibrium is maintained between the PDMS chains and low‐molecular‐weight cyclic oligosiloxanes, and the latter evaporate over time because of their relatively high vapor pressure, causing the material to deteriorate.

We focus on molecular capsules of fluoride ion (F^−^) for controlling the timing of the rearrangement of the Si–O–Si bonds. It is known that F^−^ catalyzes the rearrangement of the Si–O–Si bonds^[^ ^10^ ^]^ and that double‐four‐ring (D4R)‐type siloxane^[^ ^11^ ^]^ and germoxane (Ge–O–Ge)^[^ ^12^ ^]^ cage compounds can encapsulate F^−^. By incorporation of the molecular capsules of F^−^ into siloxane networks, the rearrangement of the Si–O–Si bonds for self‐healing can be triggered by external stimuli to release F^−^. This concept is similar to that of extrinsic self‐healing systems^[^ ^13^ ^]^ in which healing agents in microcapsules are dispersed in a matrix and released at the damaged area. The essential difference lies in the capsule sizes and release mechanisms. The D4R‐type siloxane and germoxane compounds are quite small (< 1 nm) so that transparent materials with no light scattering can be obtained. The encapsulated F^−^ can be released not by mechanical rupture, but by hydrolytic cleavage of the cage frameworks. The germoxane cage is superior to the siloxane cage because Ge–O–Ge bonds are more susceptible to hydrolysis.^[^ ^14^ ^]^ When the material is damaged, the D4R cages near the cut or cracked surfaces are exposed to moisture in the air and release F^−^ to induce local rearrangement of the siloxane networks. This can be regarded as a combined extrinsic and intrinsic healing system.

To the best of our knowledge, silicone‐based self‐healing materials containing F^−^ have not been reported, probably because of the difficulty in uniformly incorporating F^−^ into relatively hydrophobic and nonpolar networks. The D4R‐type germoxane cage can act not only as a capsule of F ^−^ but also as cross‐linkers of PDMS because of the eight functional groups radially arranged from the cage. We recently reported the functionalization of the corner Ge–OH groups of F^−^‐encapsulating germoxane cages with dimethylvinylsilyl groups.^[^ ^15^ ^]^ This allowed post‐chemical modification through hydrosilylation without deteriorating the cage structure.^[^ ^15^, ^16^ ^]^ These features of F^–^‐encapsulating germoxane cages guarantee their uniform molecular‐level incorporation into silicone networks.

**Figure 1:**
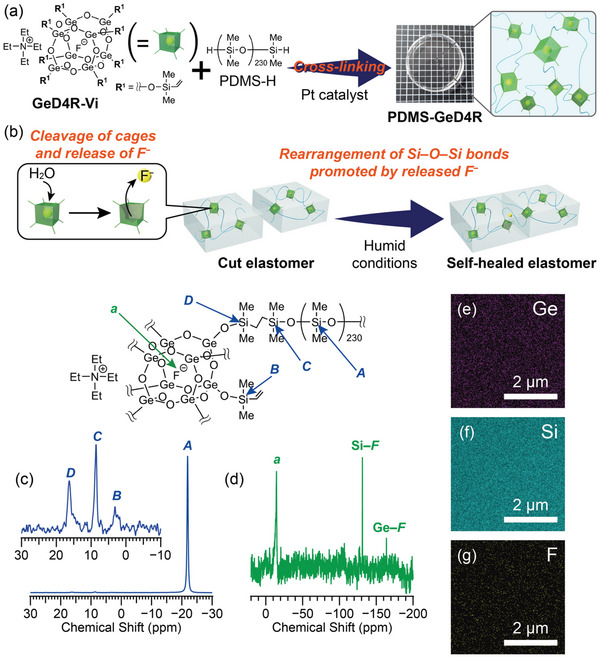
a) Synthetic scheme for self‐healable **PDMS‐GeD4R** from **GeD4R‐Vi** and PDMS‐H. b) Self‐healing of **PDMS‐GeD4R** under humid conditions. Solid‐state c) ^29^Si and d) ^19^F MAS NMR spectra, and SEM‐EDS mapping images e) Ge, f) Si, and g) F of **PDMS‐GeD4R**.

In this study, a self‐healing siloxane‐based material was designed by using F^−^‐encapsulating germoxane cages as cross‐linkers of PDMS. The hydrosilylation reaction between F^−^‐encapsulating D4R‐type germoxane cages functionalized with dimethylvinylsilyl groups (**GeD4R‐Vi**) and H‐terminated PDMS (PDMS‐H) produced a transparent elastomer (**PDMS‐GeD4R**) (**Figure** **1**a). The cut elastomers healed under humid conditions, which was triggered by the cleavage of the germoxane cages to release F^−^ (Figure 1b). For investigation of the healing mechanism, we prepared two types of F^−^‐free samples where PDMS chains are cross‐linked with either D4R siloxane cages or GeO_4_ units. A comparison of the healing efficiencies of these samples with that of **PDMS‐GeD4R** indicated that the rearrangement of the Si–O–Si networks catalyzed by F^–^ played a crucial role in rejoining of the cut surfaces.

## 2 Results and Discussion

**GeD4R‐Vi,** containing a fluoride anion inside and a tetraethylammonium cation outside the cage, was synthesized according to our previous report^[^ ^15^ ^]^ (for details, see Procedures S1 and S2 and Figures S1 and S2, Supporting Information). **GeD4R‐Vi** and PDMS‐H (average molecular weight = 17200) were dissolved in toluene and reacted in the presence of Karstedt's catalyst. After stirring at room temperature for 10 min, the reaction mixture was poured into a cylindrical perfluoroalkoxy alkane vial and allowed to stand at 60 °C for 2 d in an open system under nitrogen atmosphere. The removal of the remaining solvent under reduced pressure produced a clear and colorless elastomer (**PDMS‐GeD4R**, Figure 1a, see also Procedure S3, Supporting Information).

The solid‐state ^29^Si magic angle spinning (MAS) nuclear magnetic resonance (NMR) spectrum of **PDMS‐GeD4R** is shown in Figure 1c. The absence of the signal for the terminal SiMe_2_H groups of PDMS (≈−7 ppm) and the appearance of the signals for (SiO)*Si*Me_2_CH_2_– (**C**: *δ* = 8.6 ppm) and (GeO)*Si*Me_2_CH_2_– (**D**: *δ* = 16.3 ppm)^[^ ^16^ ^]^ indicated that hydrosilylation reaction proceeded. Signals for the residual vinylsilyl groups [(GeO)*Si*Me_2_CH=CH_2_] (**B**: *δ* = 3.1 ppm) and PDMS main chains [*Si*Me_2_(OSi)_2_] (**A**: *δ* = −21.9 ppm) were also observed. The integral ratio of the signals **D** to (**B** + **D**) indicated that 66% of the vinyl groups of **GeD4R‐Vi** (on average, 5.2 per a cage) were connected to the PDMS chains. The solid‐state ^13^C MAS NMR spectrum of **PDMS‐GeD4R** (Figure S3, Supporting Information) showed a large signal of –Si*C*H_3_ (*δ* = 0.29 ppm). Signals for –Si(*C*H_2_)_2_Si– (*δ* = 9.0 ppm), N(*C*H_2_ *C*H_3_)_4_ (*δ* = 7.0 and 52.1 ppm), and residual –Si*C*H=*C*H_2_ (*δ* = 130–140 ppm) were also observed.

The solid‐state ^19^F MAS NMR spectrum of **PDMS‐GeD4R** (Figure 1d) showed a signal at *δ* = −14.5 ppm, which was assigned to F^−^ inside the germoxane cage.^[^ ^12^, ^15^, ^16^ ^]^ Other signals at *δ* = −131.2 and −163.9 ppm were assignable to the Si–F and Ge–F species, respectively, based on the ^19^F NMR spectra of F^−^‐treated siloxane and germoxane compounds (for details, see Figures S4–S6, Supporting Information). These species indicated that F^−^ was partially released from the cage during the synthesis of **PDMS‐GeD4R**. The integral ratio of the ^19^F signal for encapsulated F^−^ to that for Si–F/Ge–F was 0.83:0.17, indicating that 83% of F^−^ were still inside the germoxane cages. The partial release of F^−^ was probably due to the cleavage of the germoxane cage by a small amount of water in the solvent or by the moisture in the air. The scanning electron microscopy (SEM)‐energy‐dispersive X‐ray spectroscopy (EDS) mapping images of **PDMS‐GeD4R** (Figures 1e,f,g) revealed uniform elemental distributions of Ge, Si, and F, indicating that the germoxane cages and encapsulated F^−^ were well‐dispersed in the siloxane networks.

**Figure 2:**
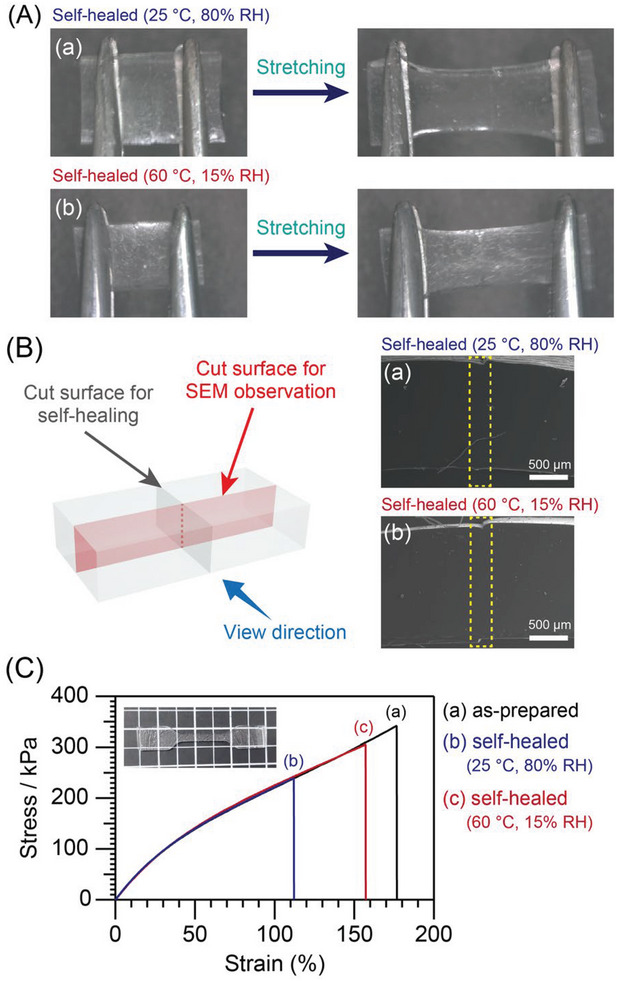
A) Product appearances and B) cross‐sectional SEM images of **PDMS‐GeD4R** after cutting and healing at a) 25 °C and 80% RH for 3 d and b) 60 °C and 15% RH for 1 d. The yellow dashed frames in the SEM images mark the self‐healed cut surfaces. C) Stress−strain curves of (a) as‐prepared **PDMS‐GeD4R** and self‐healed **PDMS‐GeD4R** treated at (b) 25 °C and 80% RH for 3 d and c) 60 °C and 15% RH for 1 d and a dumbbell‐shaped piece of **PDMS‐GeD4R** used for the tensile test (inset).

An elastomer with a thickness of ≈1.5 mm was cut into a rectangular shape and then cut in half using a razor blade to evaluate its self‐healing ability. The cut pieces were placed together and treated under humid conditions to cleave the germoxane cages near the cut surfaces. After 3 d at 25 °C and 80% RH, the cut surfaces appeared to rejoin and did not separate when pulled apart with tweezers (**Figure** **2**A(a)). The healed elastomer was cut vertically across the rejoined cut surfaces, and the cross‐section was observed by SEM. No gap was observed between the rejoined cut surfaces from the surface to the interior (Figure 2B(a)). When the cut elastomer was treated at a higher temperature with the same amount of water vapor (60 °C and 15% RH), healing occurred within a shorter time (1 d) than that required at 25 °C (3 d) (Figure 2A(b) and 2B(b)). The cut **PDMS‐GeD4R** was not self‐healed under a low humidity condition using a drying oven even if heated at 80 °C (data not shown).

Tensile tests were performed to evaluate the healing efficiency of **PDMS‐GeD4R**. The stress–strain curves of dumbbell‐shaped pieces of the as‐prepared and healed elastomers (at 25 °C and 80% RH and at 60 °C and 15% RH) are shown in Figure 2C. The stiffness of the elastomer, as indicated by the slope of the stress−strain curve, was unchanged before and after healing, suggesting that the elastomer was not deteriorated by the treatment under humid conditions. The maximum stress, maximum strain, and average healing efficiency values calculated from the maximum stresses before and after self‐healing are listed in **Table** **1**. Comparison of the maximum stress values before and after healing revealed that the average healing efficiencies at 25 °C and 80% RH and at 60 °C and 15% RH were ≈72% and 77%, respectively. The results suggest that the cut surfaces are not merely attached by noncovalent interactions such as van der Waals force but are rejoined at the molecular level by the rearrangement of the siloxane networks. It should be noted here that the healing efficiency varied from ≈50% to ≈90% depending on each sample piece tested (see Table S1, Supporting Information). The low efficiency values (≈50%) should be due to the misalignment between the attached cut surfaces, causing (i) decrease in the cross‐sectional area of the sample piece at the healed region and (ii) tearing from the steps on the elastomer surface by the stress concentration upon tensile test.

**Table 1:** Maximum stress, maximum strain, and healing efficiency values of as‐prepared and self‐healed samples

Sample	Maximum stress / kPa^a)^	Maximum strain [%]^a)^	Healing efficiency [%] ^a)^, ^b)^
As‐prepared **PDMS‐GeD4R**	302 (± 59)	181 (± 37)	–
Self‐healed **PDMS‐GeD4R** (25 °C and 80% RH for 3 d)	212 (± 45)	102 (± 44)	72.1 (± 11.0)
Self‐healed **PDMS‐GeD4R** (60 °C and 15% RH for 1 d)	236 (± 60)	132 (± 46)	76.5 (± 17.4)

^a)^ Numbers in parentheses denote standard deviations. The numbers of the measurements for as‐prepared **PDMS‐GeD4R**, self‐healed **PDMS‐GeD4R** (25 °C and 80% RH for 3 d), and self‐healed **PDMS‐GeD4R** (60 °C and 15% RH for 1 d) were 12, 6, and 7 times, respectively.

^b)^ Based on a previous report,^[^ ^17^ ^]^ the healing efficiency was calculated as the ratio of the maximum stress of the healed samples to that of the as‐prepared samples.

Tracking of the healing process showed that the healing efficiencies were 37%, 57%, 77%, and 90% after treatment at 60 °C and 15% RH for 1, 3, 6, and 24 h, respectively, and the cross‐sectional SEM images showed no gaps between the attached cut surfaces (Figure S7, Supporting Information). Thus, the cut surfaces are healed in the early stages, and the healing efficiency increased with time. Furthermore, a similarly high healing efficiency (84% on average for three samples, Table S2, Supporting Information) was obtained when the healed sample was cut again at a different position (≈300 µm away from the first cut) (Figure S8, Supporting Information) and treated at 60 °C and 15% RH for 1 d, suggesting that the healing of **PDMS‐GeD4R** was repeatable.

The ^19^F MAS NMR spectrum of **PDMS‐GeD4R** treated at 60 °C and 15% RH for 1 d (see Figure S9, Supporting Information) showed signals assigned to encapsulated F^–^, Ge–F, and Si–F. The integral ratio indicated that the remaining encapsulated F^–^ was 39%, which was lower than that before the treatment (83%). Water vapor was assumed to diffuse from the surface of the cut gel, promoting the cleavage of the cages and release of F^–^. The released F^–^ reacted not only with the Ge species, but also with the PDMS chains to induce the rearrangement of the siloxane chains.

Because the amount of germoxane cage contained in **PDMS‐GeD4R** was too small to spectroscopically investigate the cleavage behavior of the cage under humid conditions, we prepared a similar elastomer by the reaction of **GeD4R‐Vi** with H‐terminated PDMS with a lower molecular weight (consisting mainly of linear hexasiloxane) to significantly increase the germoxane content (see Procedure S4, Supporting Information). The resulting clear solid named **D6‐GeD4R** (Figure S10A, Supporting Information) was treated at 60 °C and 80% RH for 1 d. Note that the humidity was set higher than that for the self‐healing experiment to accelerate the reaction with moisture.

The ^29^Si MAS NMR spectra of **D6‐GeD4R** before and after the treatment at 60 °C and 80% RH (Figure S10B, Supporting Information) were almost the same, indicating that the main siloxane networks were retained. The ^19^F MAS NMR spectrum (Figure S10C) revealed a decrease in encapsulated F^−^ and increase in Si–F and Ge–F species. After the treatment, the FT‐IR spectrum (Figure S10D, Supporting Information) showed that the Ge–O–Ge stretching band of D4R germoxane units (≈910 cm^−1^)^[^ ^15^ ^]^ significantly decreased. Another Ge–O–Ge stretching‐derived band at lower wavenumbers (≈868 cm^−1^) assignable to cleaved germoxane cages^[^ ^15^ ^]^ increased. The decrease in the intensity of the O–Ge–O bending of D4R units (≈496 cm^−1^)^[^ ^18^ ^]^ was also observed. In the Raman spectrum (Figure S10E, Supporting Information), the intensity of the peak assigned to the four‐membered germoxane ring^[^ ^19^ ^]^ (≈420 cm^−1^) decreased after the treatment. These spectroscopic results indicate that the germoxane cages are cleaved under humid conditions, and the released F^−^ reacts with Si and Ge species.

For detailed investigation of the healing mechanism, we prepared a F^−^‐free siloxane‐based elastomer (**PDMS‐SiD4R**) using a F^−^‐free siloxane cage (**SiD4R‐Vi**) instead of **GeD4R** as a cross‐linker (see Procedure S5 and Figure S11A, Supporting Information). The healing efficiency of **PDMS‐SiD4R** after treatment at 60 °C and 15% RH for 1 d was as low as 30.9% (Figure S11B, Supporting Information). The cut surfaces were still observable in the cross‐sectional SEM image, and the sample easily separated when pulled apart with tweezers (Figure S11C and D, Supporting Information). It was assumed that the cut surfaces were physically adhered and did not heal at the molecular level. When a tetrahydrofuran (THF) solution of tetrabutylammonium fluoride (TBAF) was dropped onto the cut surface, **PDMS‐SiD4R** was repaired (Figure S11E, Supporting Information). These results indicate that the released F^–^ from the germoxane cage significantly contributes to self‐healing.

Although TBAF was effective in healing cut elastomers as described above, incorporating TBAF into the elastomer to impart self‐healing ability was difficult. For example, when **PDMS‐SiD4R** was swollen with a THF solution of TBAF and subsequently dried (Procedure S6, Supporting Information), no self‐healing behavior was observed after cutting (Figure S12A, Supporting Information). The hydrophobic PDMS networks likely hindered the impregnation of TBAF, as suggested by the SEM‐EDS analysis (Figure S12B, Supporting Information). We also attempted to introduce TBAF during the cross‐linking reactions between **SiD4R‐Vi** and PDMS‐H (Procedure S6, Supporting Information). However, the resulting elastomer exhibited a lower maximum stress (Figure S12E, Supporting Information), suggesting that TBAF inhibited hydrosilylation, as reported previously.^[^ ^8e^ ^]^ Furthermore, cut pieces were not rejoined, possibly due to phase separation of hydrated TBAF in the elastomer (Figure S12C and D (c), Supporting Information). These results highlight the effectiveness of our concept based on encapsulation and uniform distribution of F^–^ using the molecular cages.

The dehydration condensation of Ge–OH and Si–OH groups generated by the cleavage of the Ge–O–Ge and Ge–O–Si bonds might be involved in rejoining the cut surfaces. To evaluate the contribution of the Ge–OH groups to the healing efficiency, we prepared a F^−^‐free elastomer containing Ge–O–Si bonds (**PDMS‐GeO_4_**) via the dealcoholization reaction between Si–OH terminated PDMS and Ge(OEt)_4_ (Procedure S7 and Figure S13, Supporting Information). The Ge–O–Si bond is known to be hydrolytically unstable as is the Ge–O–Ge bond.^[^ ^14^ ^]^ Cut pieces of **PDMS‐GeO_4_** were placed together and treated at 60 °C and 15% RH for 1 d. However, the cut pieces were not rejoined but easily separated when pulled apart with tweezers. Thus, the contribution of the Ge–O–Si bonds to healing was negligible.

**Figure 3:**
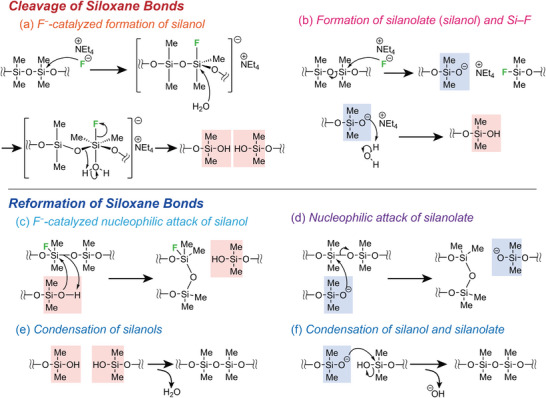
F^−^‐catalyzed cleavage and reformation of Si–O–Si bonds for self‐healing.

**Figure** **3** shows the proposed reaction mechanisms during healing based on the rearrangement of the Si–O–Si networks. First, the germoxane cages cleave upon hydrolysis of the Ge–O–Ge bonds to release F^−^. Second, the following two types of cleavage of the siloxane bonds in PDMS occur: i) the coordination of F^−^ to silicon^[^ ^10^, ^20^ ^]^ promotes nucleophilic attack by H_2_O, resulting in the formation of Si–OH end groups (Figure 3a), and ii) the nucleophilic attack of F^−^ on the silicon atoms directly forms Si–O^−^ and Si–F end groups (Figure 3b). Si–OH groups can be subsequently formed by the attack of the Si–O^−^ groups on H_2_O (Figure 3b).^[^ ^21^ ^]^

The next step is the reformation of the siloxane bonds. The nucleophilic attack of the Si–OH groups on the F^−^‐coordinated silicon atoms in other PDMS chains^[^ ^10^, ^20^ ^]^ results in the exchange of Si–O–Si bonds (Figure 3c). The Si–O^−^ groups can also contribute to the exchange reactions (Figure 3d).^[^ ^8^, ^21^, ^22^ ^]^ In addition, condensation between two Si–OH groups (Figure 3e) and Si–OH and Si–O^−^ groups (Figure 3f) occur.^[^ ^10a^ ^]^

A problem in the applications of silicone elastomers is the volatilization of low‐molecular‐weight oligomers, which, for example, causes electrical contact failure.^[^ ^23^ ^]^ Notably, during heating in air at 105 °C for 24 h, **PDMS‐GeD4R** exhibited a very small weight loss of ≈0.03%, which was smaller than the reported value for self‐healing PDMS elastomers containing silanolate end groups (≈0.15%).^[^ ^8d^ ^]^ A similarly small weight loss was observed for the sample treated at 60 °C and 15% RH for 1 d followed by air‐drying at 105 °C. We also confirmed that **PDMS‐GeD4R** showed self‐healing ability even after heating at 185 °C for 1 d (Figure S14, Supporting Information). In contrast, tetramethylammonium silanolate and potassium silanolate were reported to deactivate at temperatures higher than 120 and 170 °C, respectively.^[^ ^8c^ ^]^ Such a difference can be attributed to the higher thermal stability of the F^−^‐encapsulating cage structures and Si–F bonds over silanolates. We have previously synthesized F^−^‐encapsulating cage germoxane‐based cross‐linked networks^[^ ^16a^ ^]^ and ionic liquids^[^ ^16b^ ^]^ and investigated their thermal stability, confirming that they were thermally stable without decomposition up to ≈200 °C. The thermal and chemical stability of Si–F compounds such as SiF_4_ was also revealed by computational simulations.^[^ ^24^ ^]^ These results show that **PDMS‐GeD4R** has a significant advantage for practical applications.

## 3 Conclusion

We prepared a self‐healing siloxane‐based elastomer (**PDMS‐GeD4R**) by cross‐linking PDMS with F^−^‐encapsulating cage‐type germoxane. The germoxane cages acted not only as capsules of fluoride ions, but also as cross‐linking sites. Humid conditions promoted the cleavage of Ge–O–Ge bonds and release of fluoride ions. The released fluoride ions triggered the rearrangement of the siloxane networks, resulting in the self‐healing of the cut sample. **PDMS‐GeD4R** remained self‐healing even after repeated cutting of the elastomer. We also confirmed that the self‐healing elastomer containing the F^−^‐encapsulating germoxane cage had higher thermal stability than previously reported PDMS‐based self‐healing elastomers. Comparison with fluoride‐free samples revealed that the F^−^ in the elastomer dominantly contributed to self‐healing. Improvement of the self‐healing ability and mechanical properties of this material will be possible by changing the cage‐type germoxane content and the cross‐linking density. Thus, a new concept of healing mechanism in response to damage can be realized through the introduction of well‐dispersed cleavable molecular capsules into polymer materials.

## Conflict of Interest

The authors declare no conflict of interest.

## Author Contributions

M.S. and T. Hayashi contributed equally to this work. A.S. designed the research. M.S. and T. Hayashi performed the experiments, analyzed data, and wrote the manuscript. T. Hikino, M.K., and T.M. provided scientific and technical support. H.W., K.K., and A.S. supervised the research. All authors discussed the results and approved the final manuscript.

## Supporting information

Supporting Information

## Data Availability

The data that support the findings of this study are available from the corresponding author upon reasonable request.

## References

[1] a) S. K. Ghosh , in Self‐Healing Materials: Fundamentals, Design Strategies, and Applications, Wiley‐VCH, Weinheim 2009;

[2] P. Mazurek , S. Vudayagiri , A. L. Skov , Chem. Soc. Rev. 2019, 48, 1448. 30741275 10.1039/c8cs00963e

[3] a) S. Utrera‐Barrios , R. Verdejo , M. A. López‐Manchado , M. Hernández Santana , Mater. Horiz. 2020, 7, 2882; 10.1039/d3mh01312j 37997164

[4] a) D.‐D. Zhang , Y.‐B. Ruan , B.‐Q. Zhang , X. Qiao , G. Deng , Y. Chen , C.‐Y. Liu , Polymer 2017, 120, 189;

[5] a) F. B. Madsen , L. Yu , A. L. Skov , ACS Macro Lett. 2016, 5, 1196; 35614744 10.1021/acsmacrolett.6b00662

[6] a) J. Zhao , R. Xu , G. Luo , J. Wu , H. Xia , J. Mater. Chem. B 2016, 4, 982; 32263171 10.1039/c5tb02036k

[7] a) C.‐H. Li , C. Wang , C. Keplinger , J.‐L. Zuo , L. Jin , Y. Sun , P. Zheng , Y. Cao , F. Lissel , C. Linder , X.‐Z. You , Z. Bao , Nat. Chem. 2016, 8, 618; 27219708 10.1038/nchem.2492

[8] a) P. Zheng , T. J. McCarthy , J. Am. Chem. Soc. 2012, 134, 2024; 22280441 10.1021/ja2113257

[9] Y. Hou , G. Zhu , J. Cui , N. Wu , B. Zhao , J. Xu , N. Zhao , J Am Chem Soc 2022, 144, 436. 34965113 10.1021/jacs.1c10455

[10] a) C. J. Brinker , G. W. Scherer , in Sol‐Gel Science: The Physics and Chemistry of Sol‐Gel Processing, Academic Press, San Diego 1990;

[11] a) A. R. Bassindale , M. Pourny , P. G. Taylor , M. B. Hursthouse , M. E. Light , Angew. Chem., Int. Ed. 2003, 42, 3488; 10.1002/anie.200351249 12900960

[12] a) L. A. Villaescusa , P. Lightfoot , R. E. Morris , Chem. Commun. 2002, 2220; 10.1039/b207374a 12397987

[13] a) S. R. White , N. R. Sottos , P. H. Geubelle , J. S. Moore , M. R. Kessler , S. R. Sriram , E. N. Brown , S. Viswanathan , Nature 2001, 409, 794; 11236987 10.1038/35057232

[14] P. Eliášová , M. Opanasenko , P. S. Wheatley , M. Shamzhy , M. Mazur , P. Nachtigall , W. J. Roth , R. E. Morris , J. Čejka , Chem. Soc. Rev. 2015, 44, 7177. 25946705 10.1039/c5cs00045a

[15] N. Sato , T. Hayashi , K. Tochigi , H. Wada , A. Shimojima , K. Kuroda , Chem. Eur. J. 2019, 25, 7860. 30817031 10.1002/chem.201900439

[16] a) T. Hayashi , N. Sato , H. Wada , A. Shimojima , K. Kuroda , Dalton Trans. 2021, 50, 8497; 34047738 10.1039/d1dt01122g

[17] P. S. Tan , A. A. Somashekar , P. Casari , D. Bhattacharyya , Compos. Struct. 2019, 208, 367.

[18] a) C. S. Blackwell , J. Phys. Chem. 1979, 83, 3251;

[19] a) X. Liu , Y. Luo , W. Mao , J. Jiang , H. Xu , L. Han , J. Sun , P. Wu , Angew. Chem., Int. Ed. 2020, 59, 1166; 10.1002/anie.201912488 31674090

[20] a) T. Montheil , C. Echalier , J. Martinez , G. Subra , A. Mehdi , J. Mater. Chem. B 2018, 6, 3434; 32254441 10.1039/c8tb00456k

[21] a) A. N. Kornev , T. A. Chesnokova , V. V. Semenov , Y. A. Kurskii , Russ. Chem. Bull. 1995, 44, 1107;

[22] T. Köhler , A. Gutacker , E. Mejía , Org. Chem. Front. 2020, 7, 4108.

[23] M. Takahashi , in Organosilicon Chemistry III: From Molecules to Materials (Eds: N. Auner , J. Weis ), Wiley‐VCH, Weinheim 1997, pp. 555‐565.

[24] a) A.‐j. Tang , Q.‐s. Huan , S.‐y. Tang , D.‐j. Wei , J.‐j. Guo , Y.‐h. Zhao , RSC Adv. 2021, 11, 21832;. 35478778 10.1039/d1ra03526f PMC9034129

[25] C. Izutani , D. Fukagawa , M. Miyasita , M. Ito , N. Sugimura , R. Aoyama , T. Gotoh , T. Shibue , Y. Igarashi , H. Oshio , J. Chem. Educ. 2016, 93, 1667.

