# Supplementary material for: Integrated Extrinsic and Intrinsic Self‐Healing of Polysiloxane Materials by Cleavable Molecular Cages Encapsulating Fluoride Ions
Source: Adv Sci (Weinh). 2023 Jul 28;10(27):2303655. doi: 10.1002/advs.202303655 (PMC10520642; doi:10.1002/advs.202303655)
Supplement: Supplementary file 1 — Supporting Information [file ADVS-10-2303655-s001.pdf]

ADVANCED SCIENCE
Open Access

Supporting Information

for *Adv. Sci.*, DOI 10.1002/advs.202303655

Integrated Extrinsic and Intrinsic Self-Healing of Polysiloxane Materials by Cleavable Molecular Cages Encapsulating Fluoride Ions

*Mai Suzuki, Taiki Hayashi, Takuya Hikino, Masafumi Kishi, Takamichi Matsuno, Hiroaki Wada, Kazuyuki Kuroda and Atsushi Shimojima**

ADVANCED SCIENCE
Open Access

Supporting Information

for *Adv. Sci.*, DOI 10.1002/advs.202303655

Integrated Extrinsic and Intrinsic Self-Healing of Polysiloxane Materials by Cleavable Molecular Cages Encapsulating Fluoride Ions

*Mai Suzuki, Taiki Hayashi, Takuya Hikino, Masafumi Kishi, Takamichi Matsuno, Hiroaki Wada, Kazuyuki Kuroda and Atsushi Shimojima**

Supporting Information

Integrated Extrinsic and Intrinsic Self-Healing of Polysiloxane Materials by Cleavable Molecular Cages Encapsulating Fluoride Ions

Mai Suzuki,+[a] Taiki Hayashi,+[a] Takuya Hikino,[b] Masafumi Kishi,[a]
Takamichi Matsuno,[a,c] Hiroaki Wada,[a,c] Kazuyuki Kuroda,[a,c] and Atsushi Shimojima*[a,c]

[a] Department of Applied Chemistry, Faculty of Science and Engineering, Waseda University, 3-4-1 Okubo, Shinjuku-ku, Tokyo 169-8555 (Japan).

[b] Department of Advanced Science and Engineering, Faculty of Science and Engineering, Waseda University, 3-4-1 Okubo, Shinjuku-ku, Tokyo 169-8555 (Japan).

[c] Kagami Memorial Research Institute for Materials Science and Technology, Waseda University, 2-8-26 Nishiwaseda, Shinjuku-ku, Tokyo 169-0051 (Japan).

[+] These authors contributed equally to this work.

Table of Contents

1. Experimental Details

Materials......3

Procedure S1. Synthesis of OH-terminated germoxane cage (**GeD4R-OH**) 3

Procedure S2. Synthesis of dimethylvinylsilyl-functionalized germoxane cage (**GeD4R-Vi**) 4

Procedure S3. Hydrosilylation reaction of PDMS-H and **GeD4R-Vi** to form **PDMS-GeD4R** 4

Procedure S4. Hydrosilylation reaction of D6 and **GeD4R-Vi** to form **D6-GeD4R**......4

Procedure S5. Hydrosilylation reaction of PDMS-H and **SiD4R-Vi** to form **PDMS-SiD4R**......4

Procedure S6. Introduction of TBAF into **PDMS-SiD4R** to impart self-healing ability......5

Procedure S7. Dealcoholization reaction of OH-terminated PDMS and $Ge(OEt)_4$ to form **PDMS-GeO$_4$**......5

Characterization 6

2. Additional Data

Figure S1. (a) Powder XRD pattern, (b) FT-IR spectrum, and (c) MALDI-TOF mass spectrum of **GeD4R-OH**.......7

Figure S2. (A) ^{1}H, (B) ^{29}Si, and (C) ^{19}F NMR spectra (in toluene-*d*$_8$), (D) ESI mass spectrum, and (E) FT-IR spectrum of **GeD4R-Vi**. (F) (a) Powder XRD pattern of **GeD4R-Vi**. (b) Simulated XRD pattern of the crystal structure of **GeD4R-Vi**......8

Figure S3. ^{13}C MAS NMR spectrum of **PDMS-GeD4R**......9

Figure S4. ^{19}F NMR spectra (in THF-*d*$_8$) of (a) a THF solution of tetrabutylammonium fluoride (TBAF) and (b) a mixture of hydroxy-terminated PDMS (PDMS-OH_4200) and a THF solution of TBAF. 10

Figure S5. ^{19}F NMR spectra (in toluene-*d*$_8$) of (a) **GeD4R-Vi**, (b) a mixture of a toluene solution of **GeD4R-Vi** and a THF solution of TBAF, (c) a THF solution of TBAF, and (d) a mixture of 1,3,-divinyl-1,1,3,3-tetramethyldisiloxane (ViTMDSO) and a THF solution of TBAF. 10

Figure S6. (A) ^{1}H and (B) ^{29}Si NMR spectra (in toluene-*d*$_8$) of (a) **GeD4R-Vi**, (b) a mixture of a toluene solution of **GeD4R-Vi** and a THF solution of TBAF, and (c) ViTMDSO. 11

The interpretation of the ^{19}F MAS NMR spectrum of PDMS-GeD4R (Figure 1d) based on the NMR spectra of F$^-$-treated siloxane and germoxane compounds (Figures S4–S6). 11

Table S1. Maximum stress and healing efficiency values of **PDMS-GeD4R**. 13

Figure S7. (Left) Stress−strain curves of **PDMS-GeD4R** before and after cutting and healing at 60 °C and 15% RH for 1, 3, 6, and 24 h. (Right) Cross-sectional SEM images of **PDMS-GeD4R** after cutting and healing at 60 °C and 15% RH for 1, 3, and 6 h. 14

Table S2. Maximum stress and healing efficiency values of as-prepared **PDMS-GeD4R**, self-healed **PDMS-GeD4R** treated at 60 °C and 15% RH for 1 d, and twice-self-healed **PDMS-GeD4R** treated at 60 °C and 15% RH for 1 d......15

Figure S8. Cross-sectional SEM image of twice-self-healed **PDMS-GeD4R**......15

Figure S9. ^{19}F MAS NMR spectra of (a) as-prepared **PDMS-GeD4R** and (b) **PDMS-GeD4R** treated at 60 °C and 15% RH for 1 d. 16

Figure S10. (A) Product appearance of **D6-GeD4R**. (B) ^{29}Si MAS NMR spectra, (C) ^{19}F MAS NMR spectra, (D) FT-IR spectra of **D6-GeD4R** (a) before and (b) after treatment at 60 °C and 80% RH for 1 d. (E) Raman spectra of **D6-GeD4R** (a) before and (b) after treatment at 60 °C and 80% RH for 1 d and (c) **GeD4R-OH**......17

Figure S11. (A) ^{29}Si MAS NMR spectrum of **PDMS-SiD4R**. (B) Stress–strain curves of **PDMS-SiD4R** (a) as-synthesized and (b) after cutting and treatment at 60 °C and 15% RH for 1 d. (C) Product appearance and (D) cross-sectional SEM image of **PDMS-SiD4R** after cutting and treatment at 60 °C and 15% RH for 1 d. (E) Product appearances of **PDMS-SiD4R** after cutting and healing at 40 °C for 1 d with a drop of THF solution of TBAF......18

Figure S12. (A) Product appearance of **PDMS-SiD4R-TBAF_1** after cutting and treatment at 60 °C and 15% RH for 1 d. (B) SEM-EDS mapping of fluorine in **PDMS-SiD4R-TBAF_1**. (C) Product appearances of **PDMS-SiD4R-TBAF_2** (left) before and (right) after cutting and treatment at 60 °C and 15% RH for 1 d. (D) Optical microscopy images of (a) **PDMS-SiD4R**, (b) **PDMS-SiD4R-TBAF_1**, and (c) **PDMS-SiD4R-TBAF_2**. (E) Stress–strain curve of **PDMS-SiD4R-TBAF_2**. 19

Figure S13. (a) FT-IR spectrum, (b) ^{29}Si MAS NMR spectrum, and (c) stress–strain curve of **PDMS-GeO$_4$**......20

Figure S14. Product appearances of heated **PDMS-GeD4R** (185 °C for 1 d) after cutting and healing at 60 °C and 15% RH for 1 d. .20

References 21

1. Experimental Details

Materials

The following reagents were used without purification: acetone (Kanto Chemical Co. Inc., >99.0%), chlorodimethylvinylsilane (ViDMSCl, Tokyo Chemical Industry Co. Ltd., >95.0%), germanium oxide (GeO_2, Kanto Chemical Co. Inc., >99.99%), hexane (Kanto Chemical Co. Inc., >95.0%), hydride terminated polydimethylsiloxane (D6, molecular weight: average 580,[1] Sigma Aldrich), hydroxy terminated polydimethylsiloxane (PDMS-OH_4200, molecular weight: average 4200, Thermo Scientific (Alfa Aesar)), hydride terminated polydimethylsiloxane (PDMS-H, molecular weight: average 17200, viscosity: 500 cSt , Gelest, Inc., >95%), magnesium sulfate (anhydrous, FUJIFILM Wako Pure Chemical Corp., >98.0%), methanol (FUJIFILM Wako Pure Chemical Corp., >99.8%), Palladium Hydroxide on Carbon (Pearlman's catalyst, Tokyo Chemical Industry Co. Ltd., wetted with ca. 50% Water), platinum(0)-1,3-divinyl-1,1,3,3-tetramethyldisiloxane complex in xylene (Karstedt's catalyst, Sigma Aldrich), tetrabutylammonium fluoride (TBAF) solution (1.0 M in tetrahydrofuran, Sigma-Aldrich), tetraethylammonium fluoride hydrate (TEAF, Tokyo Chemical Industry Co. Ltd., >98.0%), tetrahydrofuran (THF, super-dehydrated, stabilizer free, FUJIFILM Wako Pure Chemical Corp., >99.5%), 1,1,3,3-tetramethyl-1,3-divinyldisilazane (ViTMDS, Tokyo Chemical Industry Co. Ltd., >95.0%), 1,1,3,3-tetramethyl-1,3-divinyldisiloxane (ViTMDSO, Tokyo Chemical Industry Co. Ltd., >98.0%), and toluene (super-dehydrated, FUJIFILM Wako Pure Chemical Corp., >99.5%).

Tetraethoxygermane ($Ge(OEt)_4$, Gelest, inc., 97%) was purified by distillation (100 °C, reduced pressure).

Procedure S1. Synthesis of OH-terminated germoxane cage (**GeD4R-OH**)

GeD4R-OH $[Ge_8O_{12}(OH)_8F][N(C_2H_5)_4]$ was synthesized according to our previous report.[2] The content of water in TEAF hydrate was determined by the ^1H nuclear magnetic resonance (NMR) analysis of a dimethylsulfoxide-*d*$_6$ solution of TEAF hydrate containing acetonitrile as a standard substance. GeO_2 (5.53 g, 52.8 mmol), TEAF・2.5H_2O (5.01 g, 26.0 mmol), and H_2O (1.40 mL, 77.5 mmol) were mixed in a Teflon vessel and stirred by a Teflon rod. The molar ratio of GeO_2/TEAF/H_2O was 10:5:27. The mixture was heated at 150 °C for 7 d. Then, H_2O (5 mL) and methanol (5 mL) were added to the reaction mixture. After ultrasonication (35 W, 40 kHz) (USS-1, NIHONSEIKI KAISHA LTD.) for 10 min, an excess amount of acetone was added, and the mixture was further ultrasonicated. The mixture was allowed to stand for 3 d, and a white precipitate was formed. After the addition of H_2O (10 mL), methanol (10 mL), and acetone (10 mL), the precipitate was recovered by centrifugation (5000 rpm, 10 min) to obtain a white solid (4.29 g, 61% yield).

The powder X-ray diffraction (XRD) pattern (Figures S1a) and Fourier transform infrared (FT-IR) spectrum (Figures S1b) of the product were consistent with those of our previously reports.[2] The matrix-assisted laser desorption ionization time-of-flight (MALDI-TOF) mass spectrum (Figure S1c) shows the peaks corresponding to $[Ge_8O_{12}(OH)_8F]^-$ (centered at *m/z* 928). The isotope pattern of the peak was consistent with those obtained by simulation. These results indicated that **GeD4R-OH** was successfully synthesized.

Procedure S2. Synthesis of dimethylvinylsilyl-functionalized germoxane cage (**GeD4R-Vi**)

GeD4R-Vi $[Ge_8O_{12}(OSiMe_2CHCH_2)_8F][N(C_2H_5)_4]$ was synthesized according to our previous reports.[2] THF (10 mL), **GeD4R-OH** (0.60 g, 0.35 mmol), ViTMDS (2.0 mL, 0.88 mmol), and ViDMSCl (1.2 mL, 0.89 mmol) were mixed in a two-neck round-bottom flask equipped with a reflux condenser under a nitrogen atmosphere. After stirring for 1 d at 60 °C, volatile compounds were removed at 90 °C under reduced pressure. The product was dissolved in 100 mL of toluene and insoluble compounds were removed by a syringe filter (0.22 µm pore size, poly(tetrafluoroethylene membrane (PTFE)). The solvent was removed using a rotary evaporator at 40 °C. The residual product was washed with hexane and recovered by centrifugation (5000 rpm, 10 min) to obtain a pale-yellow solid (0.45 g, 45% yield). ^{1}H NMR (500.16 MHz, toluene-d_8): δ (ppm) = 0.41 (s, 48H; SiCH_3), 1.03 (br, 12H; NCH_2CH_3), 2.81 (br, 8H, NCH_2CH_3), 5.91 (dd, J = 20.1, 4.0 Hz, 8H; SiCH=CH_2), 5.99 (dd, J = 14.9, 4.0 Hz, 8H; SiCH=CH_2), 6.36 (dd, J = 20.1, 14.9 Hz, 8H; SiCH=CH$_2$); ^{19}F NMR (470.62 MHz, toluene-d_8): δ (ppm) = –12.95; ^{29}Si NMR (99.37 MHz, toluene-d_8); δ (ppm) = 2.43 (–OSiMe$_2$CHCH$_2$).

The ^{1}H, ^{29}Si, and ^{19}F NMR spectra, electrospray ionization (ESI) mass spectrum, FT-IR spectrum, and powder XRD pattern of **GeD4R-Vi** are shown in Figure S2. The ESI mass spectrum (Figure S2D) showed the peak corresponding to $[Ge_8O_{12}(OSiMe_2CH{=}CH_2)_8F]^-$ (centered at m/z 1601). The powder XRD pattern (Figure S2F(b)) was consistent with the simulation pattern obtained by single-crystal X-ray structural analysis data (Figure S2F(a)).[2a]

Procedure S3. Hydrosilylation reaction of PDMS-H and **GeD4R-Vi** to form **PDMS-GeD4R**

GeD4R-Vi (0.14 g, 0.080 mmol) and PDMS-H (2.76 g, 0.161 mmol) were dissolved in 20 mL of toluene under a nitrogen atmosphere, followed by the addition of a Karstedt’s catalyst (0.50 µL). After stirring for 10 min., the mixture was poured into a cylindrical perfluoroalkoxy alkane (PFA) vial placed in a separable flask under a nitrogen atmosphere. The mixture was kept at 60 °C for 2 d to form an elastic elastomer. The remaining solvent was removed under reduced pressure at 60 °C to obtain a clear and colorless elastomer.

Procedure S4. Hydrosilylation reaction of D6 and **GeD4R-Vi** to form **D6-GeD4R**

GeD4R-Vi (0.30 g, 0.17 mmol) and D6 (0.17 g, 0.35 mmol) were dissolved in 30 mL of toluene under a nitrogen atmosphere. Then, Karstedt’s catalyst (1 µL) was added. After stirring for 10 min, the mixture was poured into a cylindrical PFA vial placed in a separable flask under a nitrogen atmosphere. The mixture in a closed vial was kept at 60 °C for 1 d. The remaining solvents were removed under reduced pressure at 60 °C to obtain a clear and colorless solid.

Procedure S5. Hydrosilylation reaction of PDMS-H and **SiD4R-Vi** to form **PDMS-SiD4R**

Dimethylvinylsilylated siloxane cage (**SiD4R-Vi**) was synthesized according to the previous report.[3] Tetramethylammonium D4R silicate ($Si_8O_{20}^-$) hydrate (5.3 g) was dissolved in methanol (7 mL). The solution was added to the mixture of ViDMSCl (10 mL) and hexane (10 mL). After stirring for 1 d at room temperature, the hexane layer was extracted and volatile compounds were removed at 40 °C under reduced pressure. The product was washed with acetonitrile to obtain a white solid (2.61 g, 93% yield). ^{1}H NMR (500.16 MHz, chloroform-d): δ (ppm) = 0.21 (s, 48H; SiCH$_3$), 5.79 (dd, J = 5.79, 20.1, 4.0 Hz, 8H; SiCH=CH_2), 5.96 (dd, J = 14.9, 4.0 Hz, 8H; SiCH=CH_2), 6.12 (dd, J = 20.1, 14.9

Hz, 8H; SiCH=CH_2); ^{29}Si NMR (99.37 MHz, chloroform-*d*); δ (ppm) = 0.52 (–O*Si*Me_2CHCH_2), −109.12 (*Si*$(OSi)_4$).

SiD4R-Vi (0.11 g, 0.086 mmol) and PDMS-H (3.01 g, 0.175 mmol) were dissolved in 20 mL of toluene under a nitrogen atmosphere, followed by the addition of a Karstedt's catalyst (0.50 µL). After stirring at room temperature for 10 min, the mixture was poured into a cylindrical PFA vial placed in a separable flask under a nitrogen atmosphere. The mixture in a closed vial was kept at 60 °C for 2 d to form an elastic elastomer. The remaining solvents were removed under reduced pressure at 60 °C to obtain a clear and colorless elastomer.

Procedure S6. Introduction of TBAF into **PDMS-SiD4R** to impart self-healing ability

The introduction of TBAF into **PDMS-SiD4R** was performed by the following two methods: (1) post-incorporation of TBAF into **PDMS-SiD4R** and (2) introduction of TBAF during the cross-linking reactions between **SiD4R-Vi** and PDMS-H to form **PDMS-SiD4R**.

(1) **PDMS-SiD4R** (0.09 g) was swollen with a mixture of THF (97.5 µL) and a TBAF solution (1.0 M in THF, 2.6 µL). After 30 min at room temperature, the elastomer was lightly rinsed with THF to remove outside TBAF and was subsequently dried under reduced pressure. The resulting elastomer is denoted as **PDMS-SiD4R-TBAF_1** in Figure S12.

(2) **SiD4R-Vi** (0.11 g, 0.086 mmol) and PDMS-H (3.13 g, 0.182 mmol) were dissolved in 20 mL of THF under a nitrogen atmosphere, followed by the addition of a Karstedt's catalyst (0.50 µL). After stirring at room temperature for 10 min, a TBAF solution (1.0 M in THF, 86 µL) was added to the reaction mixture. The mixture was poured into a cylindrical PFA vial placed in a separable flask under a nitrogen atmosphere. The mixture in a closed vial was kept at 60 °C for 2 d to form an elastomer. The resulting elastomer is denoted as **PDMS-SiD4R-TBAF_2**.

Procedure S7. Dealcoholization reaction of OH-terminated PDMS and $Ge(OEt)_4$ to form **PDMS-GeO$_4$**

First, terminal Si–H groups of PDMS-H was converted to Si–OH groups. PDMS-H (1.92 g, 0.11 mmol) was added to a two-neck round-bottom flask and dried under vacuum for 30 min. THF (20 mL) and H_2O (20 µL. 1.1 mmol) were added to the flask under a nitrogen atmosphere and cooled at 0 °C by an ice bath. Thereafter, Pearlman's catalyst (0.07 g) was added, and the mixture was stirred at 0 °C and then returned to room temperature. After stirring for 1 d, the mixture was dried with magnesium sulfate and filtrated by a PTFE membrane (0.22 µm pore size). The resulting clear solution was concentrated at room temperature under reduced pressure to obtain a clear viscous liquid. ^{1}H NMR (500.16 MHz, chloroform-*d*): δ (ppm) = 0.07 (s; SiCH_3); ^{13}C NMR (125.77 MHz, chloroform-*d*): δ (ppm) = 1.04 (SiCH_3); ^{29}Si NMR (99.37 MHz, chloroform-*d*); δ (ppm) = −10.35 (–O*Si*Me_2OH), −21.94 (–O*Si*Me_2O–).

Next, dealcoholization reaction of the resulting OH-terminated PDMS and $Ge(OEt)_4$ was conducted to form a siloxane-based elastomer containing Ge–O–Si bonds. OH-terminated PDMS was dissolved in toluene (10 mL) under a nitrogen atmosphere. Then, $Ge(OEt)_4$ (18.7 µL, 0.08 mmol) was added to the solution. After stirring at 40 °C for 30 min, the mixture was poured into a cylindrical PFA vial placed in a separable flask under a nitrogen atmosphere. The mixture in a closed vial was kept at 80 °C for 1 d to form a clear and colorless elastomer.

The FT-IR spectrum of **PDMS-GeO$_4$** (Figure S13a) showed a small band (~960 cm^{-1}) attributed to Ge–O–Si stretching.[4] In the ^{29}Si MAS NMR spectrum (Figure S13b), a small signal newly appeared at δ = −16.2 ppm may be associated with the formation of *Si*–O–Ge bonds. These results suggested the formation of a siloxane-based elastomer containing Ge–O–Si bonds.

Characterization

Liquid-state ^{1}H, ^{13}C, ^{19}F, and ^{29}Si NMR spectra were recorded on a JEOL JNM ECZ 500 spectrometer at resonance frequencies of 500.16, 125.77, 470.62, and 99.37 MHz, respectively, at ambient temperature using 5 mm glass tubes. Toluene-d_8 was used to obtain lock signals. Tetramethylsilane (TMS) (δ = 0 ppm) was used as the internal reference for ^{1}H, ^{13}C, and ^{29}Si NMR spectroscopy. Trifluoromethylbenzene (−62.88 ppm for toluene-d_8 and −63.47 ppm for THF-d_8)[5] was used as an external reference for ^{19}F NMR spectroscopy. A small amount of Cr(acac)$_3$ (acac = acetylacetonate) was used as a relaxation agent for the ^{29}Si nuclei. The ^{29}Si NMR spectra were measured with a 45° pulse and a recycle delay of 10 s. Solid-state ^{13}C magic angle spinning (MAS) NMR spectra were recorded on a JEOL JNM ECA 400 spectrometer at a resonance frequency of 100.53 MHz with a contact time of 5 ms and a recycle delay of 10 s at ambient temperature. Solid-state ^{19}F MAS NMR spectra were recorded on a JEOL JNM ECA 400 spectrometer at a resonance frequency of 376.17 MHz with a recycle delay of 5 s at ambient temperature. Solid-state ^{29}Si MAS NMR spectra were recorded on a JEOL JNM ECA 400 spectrometer at a resonance frequency of 79.43 MHz with a 90° pulse and a recycle delay of 50 s at ambient temperature. The samples for solid-state NMR analysis were put in a 5 mm zirconia rotor and spun at 8 kHz. Hexamethylbenzene (δ = 17.4 ppm), poly(tetrafluoroethylene) (δ = −124.0 ppm), and poly(dimethylsilane) (δ = –33.8 ppm) were used as external references for solid-state ^{13}C, ^{19}F, and ^{29}Si NMR spectroscopy, respectively. MALDI-TOF mass analysis was conducted using a Bruker autoflex maX instrument with 2,5-dihydroxybenzoic acid (DHB) as the matrix. ESI mass analysis was conducted using a JEOL JMS T100 CS AccuTOF instrument. FT-IR spectroscopy analysis was performed using a JASCO FT/IR-6100 spectrometer. The FT-IR spectra of the powder samples were obtained using the KBr method. The FT-IR spectra of other samples were obtained using the attenuated total reflection (ATR) method with an ATR accessory (JASCO ATR PRO ONE) with a germanium prism. Raman spectroscopy analysis was conducted using a Renishaw InVia Reflex spectrometer (excitation wavelength: 532 nm). XRD analysis was conducted using a RIGAKU Rint-Ultima III diffractometer with parallel beam geometry equipped with a scintillation detector and parabolic multilayer mirror using Cu Kα radiation (40 kV, 40 mA). Scanning electron microscopy (SEM) images were obtained using a HITACHI S-3000N at an accelerating voltage of 25 kV. All samples were sputtered with Pt before the SEM observations. The distributions of F, Si, and Ge in the SEM images were recorded by energy-dispersive X-ray spectroscopy (EDS) using a Si (Li) detector. Tensile tests were performed by a SHIMADZU Table-Top Universal Tester EZ-SX 200N. Dumbbell pieces for the tensile tests were prepared using a SD Lever Type Sample Cutting Machine SDL-100 equipped with a Super Dumbbell® cutter SDMP-1000-D (Dumbbell Co., Ltd., JIS K-6251-7). Optical microscopy images were obtained using an Olympus BX51 microscope. The temperature and humidity for the self-healing were controlled using an environmental test chamber ESPEC SH-222.

2. Additional Data

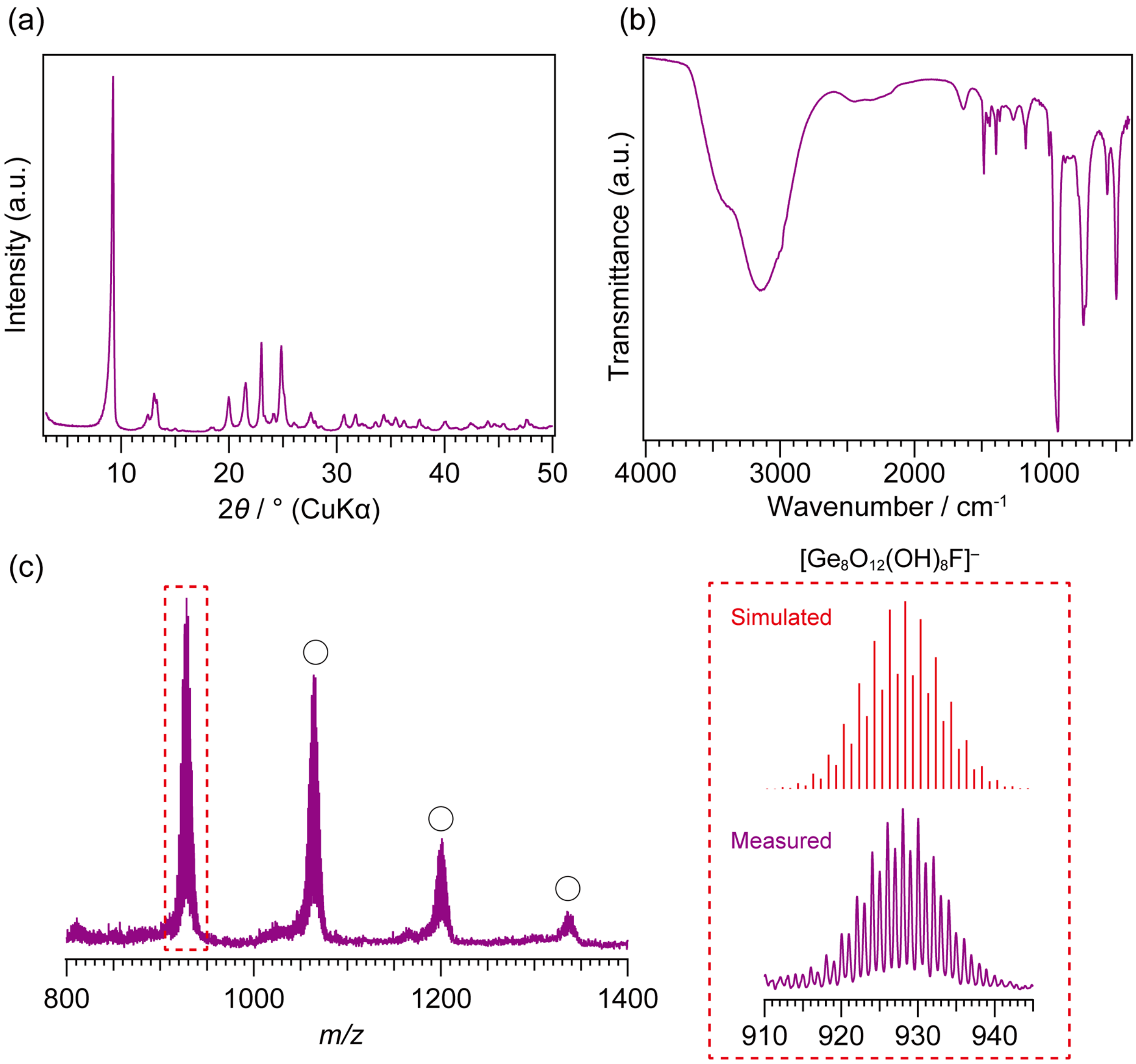

Figure S1. (a) Powder XRD pattern, (b) FT-IR spectrum, and (c) MALDI-TOF mass spectrum of **GeD4R-OH**. ○: The peaks of **GeD4R-OH** anions in which one to three of the terminal hydroxy groups was reacted with fragments of DHB used as the matrix.[6]

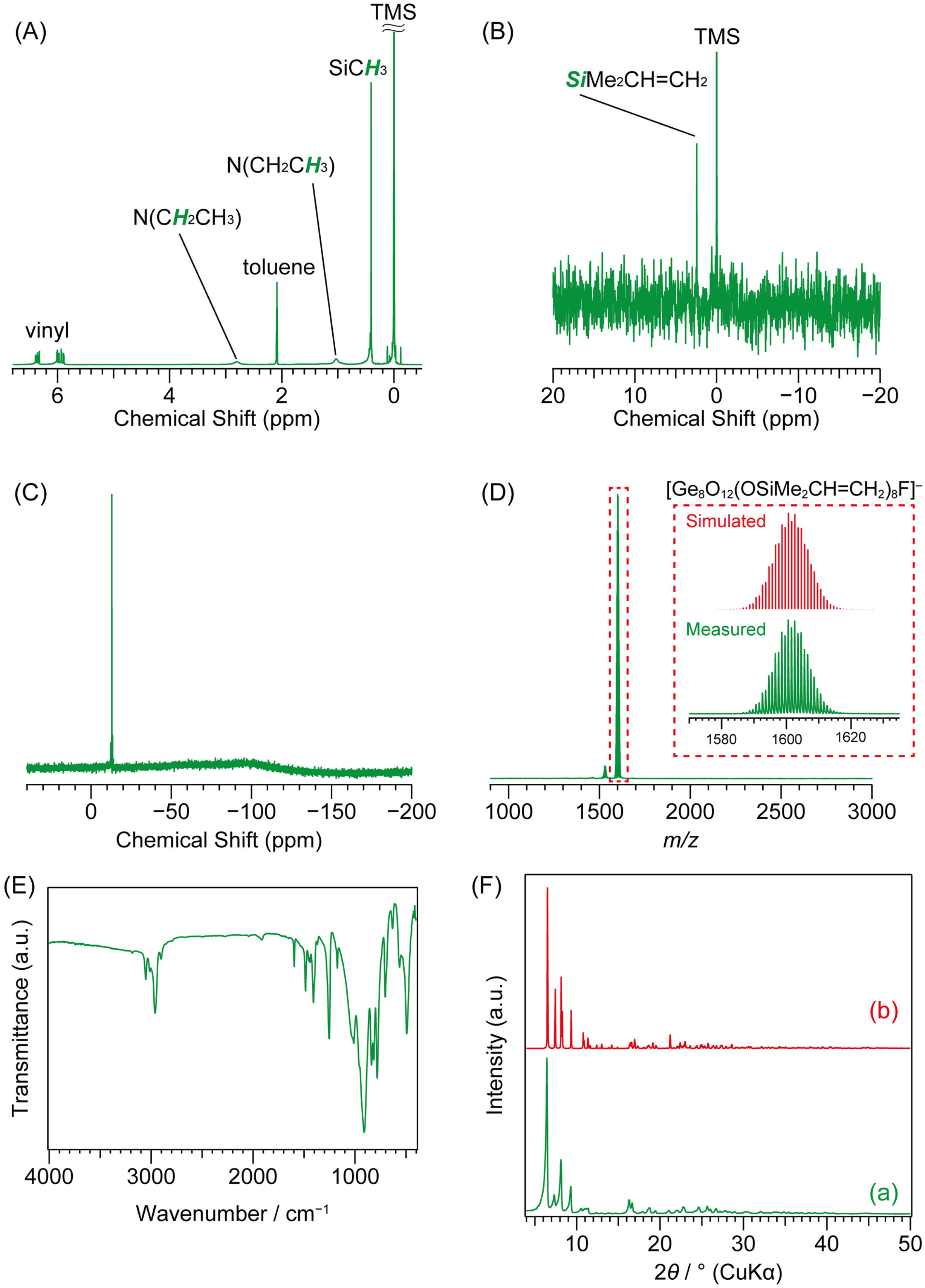

Figure S2. (A) ^{1}H, (B) ^{29}Si, and (C) ^{19}F NMR spectra (in toluene-d_8), (D) ESI mass spectrum, and (E) FT-IR spectrum of **GeD4R-Vi**. (F) (a) Powder XRD pattern of **GeD4R-Vi**. (b) Simulated XRD pattern of the crystal structure of **GeD4R-Vi**.

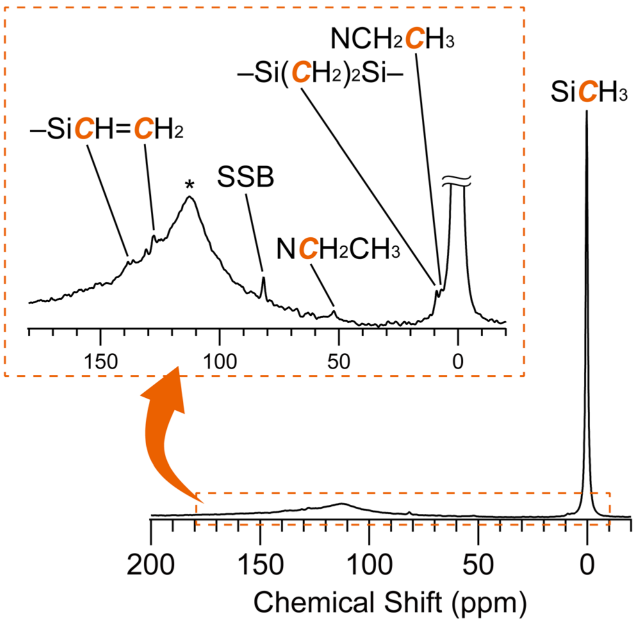

Figure S3. ^{13}C MAS NMR spectrum of **PDMS-GeD4R**. The asterisk (*) and SSB indicate the carbon background signal attributable to the probe and the spinning side band, respectively.

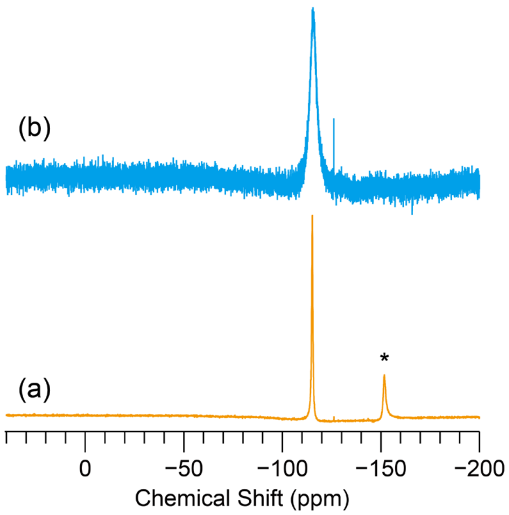

Figure S4. ^{19}F NMR spectra (in THF-d_8) of (a) a THF solution of tetrabutylammonium fluoride (TBAF) and (b) a mixture of hydroxy-terminated PDMS (PDMS-OH_4200) and a THF solution of TBAF. The asterisk (*) indicates the signal of HF_2^- generated by the decomposition of TBAF. [7]

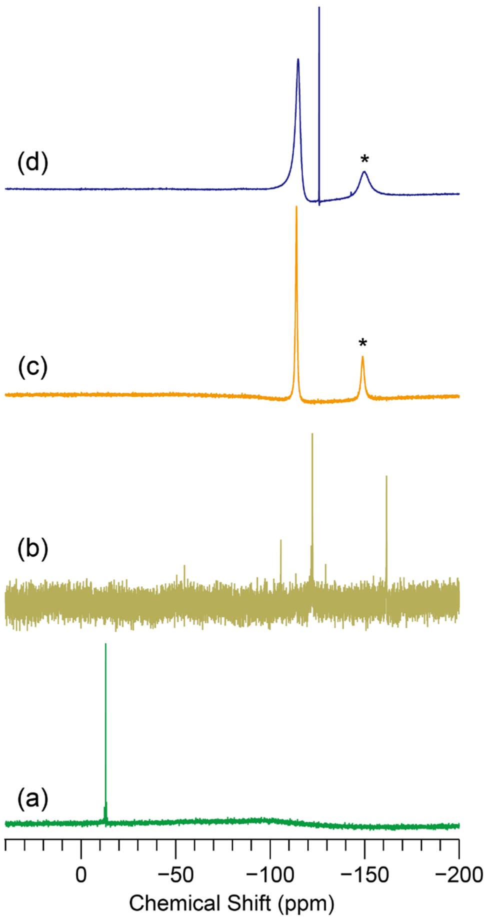

Figure S5. ^{19}F NMR spectra (in toluene-d_8) of (a) **GeD4R-Vi**, (b) a mixture of a toluene solution of **GeD4R-Vi** and a THF solution of TBAF, (c) a THF solution of TBAF, and (d) a mixture of 1,3,-divinyl-1,1,3,3-tetramethyldisiloxane (ViTMDSO) and a THF solution of TBAF. The asterisks (*) indicate the signal of HF_2^- generated by the decomposition of TBAF.[7]

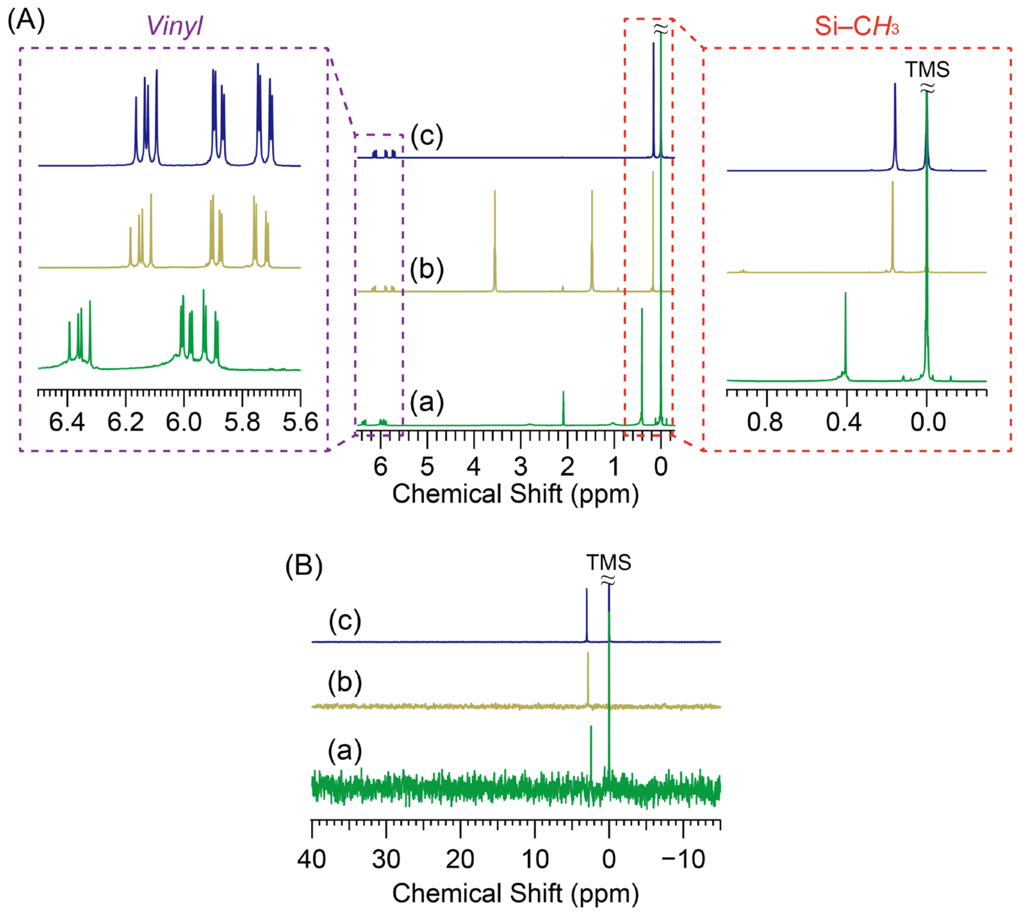

Figure S6. (A) ^{1}H and (B) ^{29}Si NMR spectra (in toluene-d_8) of (a) **GeD4R-Vi**, (b) a mixture of a toluene solution of **GeD4R-Vi** and a THF solution of TBAF, and (c) ViTMDSO.

The interpretation of the ^{19}F MAS NMR spectrum of PDMS-GeD4R (Figure 1d) based on the NMR spectra of F⁻-treated siloxane and germoxane compounds (Figures S4–S6).

The ^{19}F MAS NMR spectrum of **PDMS-GeD4R** showed the signals at δ = −14.5, −131.2, −163.9 ppm (see Figure 1d). The signal at δ = −14.5 ppm was assignable to F⁻ inside the germoxane cage.[2,8] For the assignment of the other two ^{19}F signals, we performed the NMR measurements of PDMS-OH_4200, **GeD4R-Vi**, and ViTMDSO after the treatment with TBAF.

In the ^{19}F NMR spectrum of PDMS-OH_4200 treated with a THF solution of TBAF (Figure S4), a new signal was observed at −126.10 ppm, which was presumably due to the Si–F bonds.

When a toluene solution of **GeD4R-Vi** was mixed with a THF solution of TBAF, a precipitate was formed, which was indicative of the cleavage of Ge–O–Ge and/or Ge–O–Si bonds. In the ^{19}F NMR spectrum of the solution after removal of the precipitate (Figure S5b), the signals of F⁻ inside the cages (Figure S5a) and TBAF (Figure S5c) were not observed, but new signals were mainly observed at δ = −122.32 ppm and −161.56 ppm. These signals could correspond to the fluorine species bonded to the silicon and germanium species. In the ^{1}H and ^{29}Si NMR spectra of this solution (Figure S6b), the signal of the dimethylvinylsilyl groups in **GeD4R-Vi** (Figure S6a) was not observed, but a signal assignable to ViTMDSO (Figure S6c) was observed, suggesting that the fluoride ions derived from TBAF induced the cleavage of the Si–O–Ge bonds in **GeD4R-Vi** and subsequent condensation of the

dimethylvinylsilane species. However, the signals of SiF species (δ = ~35 ppm)[9] were not detected probably due to the low concentration.

Then, we performed the ^{19}F NMR measurement of the mixture of ViTMDSO and a THF solution of TBAF. In the ^{19}F NMR spectrum (Figure S5d) a signal was observed at δ = −125.91 ppm and no signal was observed at around δ = −160 ppm. From all these results, ^{19}F signals at around δ = −130 and −160 ppm were attributed to Si–F and Ge–F bonds, respectively. This assignment was reasonable from the fact that in the ^{19}F MAS NMR spectrum of **D6-GeD4R** (a silicone network with shorter linear siloxanes and higher germoxane cage content compared to **PDMS-GeD4R**, see also Procedure S4 and Figure S10c), the signal at around δ = −160 ppm was larger than the signal at around δ = −130 ppm, unlike **PDMS-D4R**.

Table S1. Maximum stress and healing efficiency values of **PDMS-GeD4R**.

Entry	Sample	Maximum Stress (kPa)	Strain (%)	Healing efficiency (%)
1	As-prepared **PDMS-GeD4R**	414	196	-
2	As-prepared **PDMS-GeD4R**	241	141	-
3	As-prepared **PDMS-GeD4R**	267	227	-
4	As-prepared **PDMS-GeD4R**	315	191	-
	Self-healed **PDMS-GeD4R** at 25 °C and 80% RH for 3 d	241	141	76.5
5	As-prepared **PDMS-GeD4R**	225	167	-
	Self-healed **PDMS-GeD4R** at 25 °C and 80% RH for 3 d	135	38	60.2
6	As-prepared **PDMS-GeD4R**	324	251	-
	Self-healed **PDMS-GeD4R** at 60 °C and 15% RH for 1 d	194	180	60.0
7	As-prepared **PDMS-GeD4R**	225	167	-
	Self-healed **PDMS-GeD4R** at 60 °C and 15% RH for 1 d	135	38	60.2
8	As-prepared **PDMS-GeD4R**	310	201	-
	Self-healed **PDMS-GeD4R** at 60 °C and 15% RH for 1 d	281	182	90.5
9	As-prepared **PDMS-GeD4R**	303	141	-
	Self-healed **PDMS-GeD4R** at 25 °C and 80% RH for 3 d	185	68	61.0
	Self-healed **PDMS-GeD4R** at 60 °C and 15% RH for 1 d	152	52	50.2
10	As-prepared **PDMS-GeD4R**	242	145	-
	Self-healed **PDMS-GeD4R** at 25 °C and 80% RH for 3 d	216	153	89.5
	Self-healed **PDMS-GeD4R** at 60 °C and 15% RH for 1 d	218	119	90.4
11	As-prepared **PDMS-GeD4R**	339	141	-
	Self-healed **PDMS-GeD4R** at 25 °C and 80% RH for 3 d	256	98	75.7
	Self-healed **PDMS-GeD4R** at 60 °C and 15% RH for 1 d	304	125	89.9
12	As-prepared **PDMS-GeD4R**	342	177	-
	Self-healed **PDMS-GeD4R** at 25 °C and 80% RH for 3 d	240	112	70.1
	Self-healed **PDMS-GeD4R** at 60 °C and 15% RH for 1 d	305	157	89.3

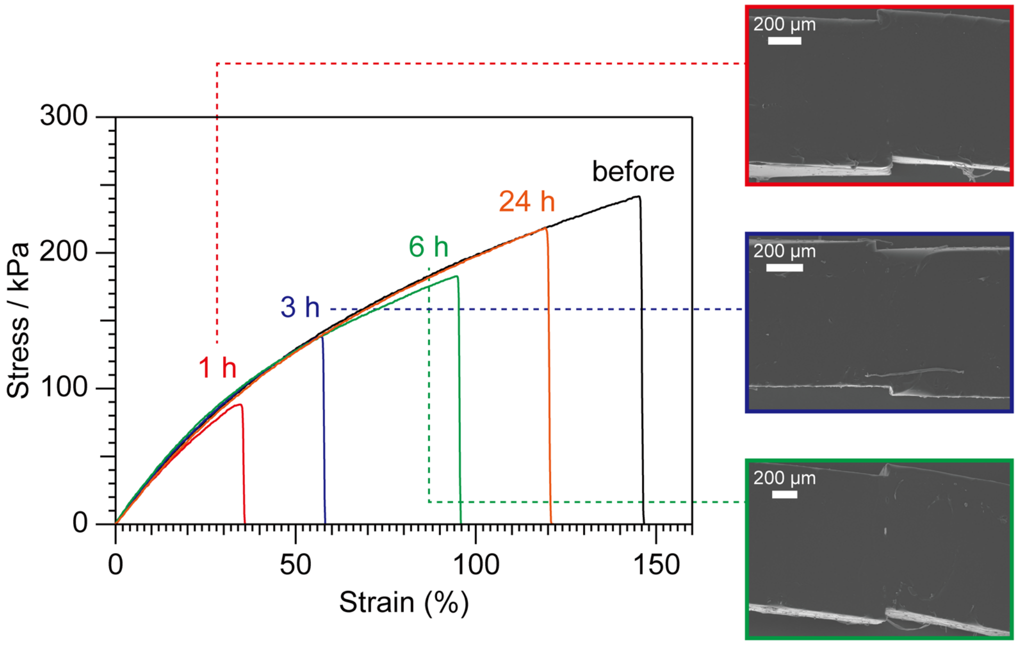

Figure S7. (Left) Stress−strain curves of **PDMS-GeD4R** before and after cutting and healing at 60 °C and 15% RH for 1, 3, 6, and 24 h. (Right) Cross-sectional SEM images of **PDMS-GeD4R** after cutting and healing at 60 °C and 15% RH for 1, 3, and 6 h. Because a total of five dumbbell pieces, which are needed to track the changes over time, could not be cut from a single elastomer, rectangular cut pieces (1 × 2 × 10 mm) were used for the tensile tests.

Table S2. Maximum stress and healing efficiency values of as-prepared **PDMS-GeD4R**, self-healed **PDMS-GeD4R** treated at 60 °C and 15% RH for 1 d, and twice-self-healed **PDMS-GeD4R** treated at 60 °C and 15% RH for 1 d.

Entry	Sample	Maximum stress (kPa)	Healing efficiency (%)
1	As-prepared **PDMS-GeD4R**	315	-
	Self-healed **PDMS-GeD4R**	260	82.5
	Twice-self-healed **PDMS-GeD4R**	263	83.5
2	As-prepared **PDMS-GeD4R**	310	-
	Self-healed **PDMS-GeD4R**	260	83.9
	Twice-self-healed **PDMS-GeD4R**	281	90.6
3	As-prepared **PDMS-GeD4R**	342	-
	Self-healed **PDMS-GeD4R**	305	89.2
	Twice-self-healed **PDMS-GeD4R**	268	78.4

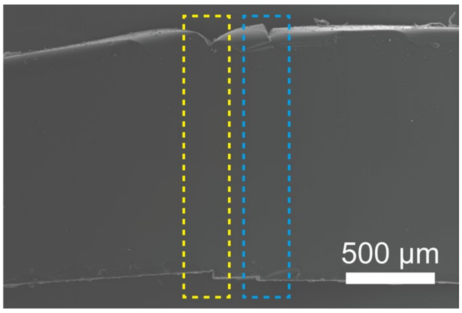

Figure S8. Cross-sectional SEM image of twice-self-healed **PDMS-GeD4R**. The yellow and light blue dashed frame in the SEM image marks the cut surface.

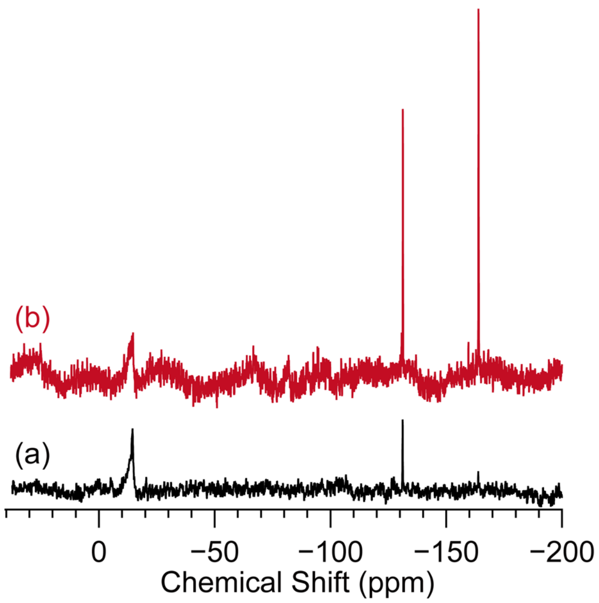

Figure S9. ^{19}F MAS NMR spectra of (a) as-prepared **PDMS-GeD4R** and (b) **PDMS-GeD4R** treated at 60 °C and 15% RH for 1 d.

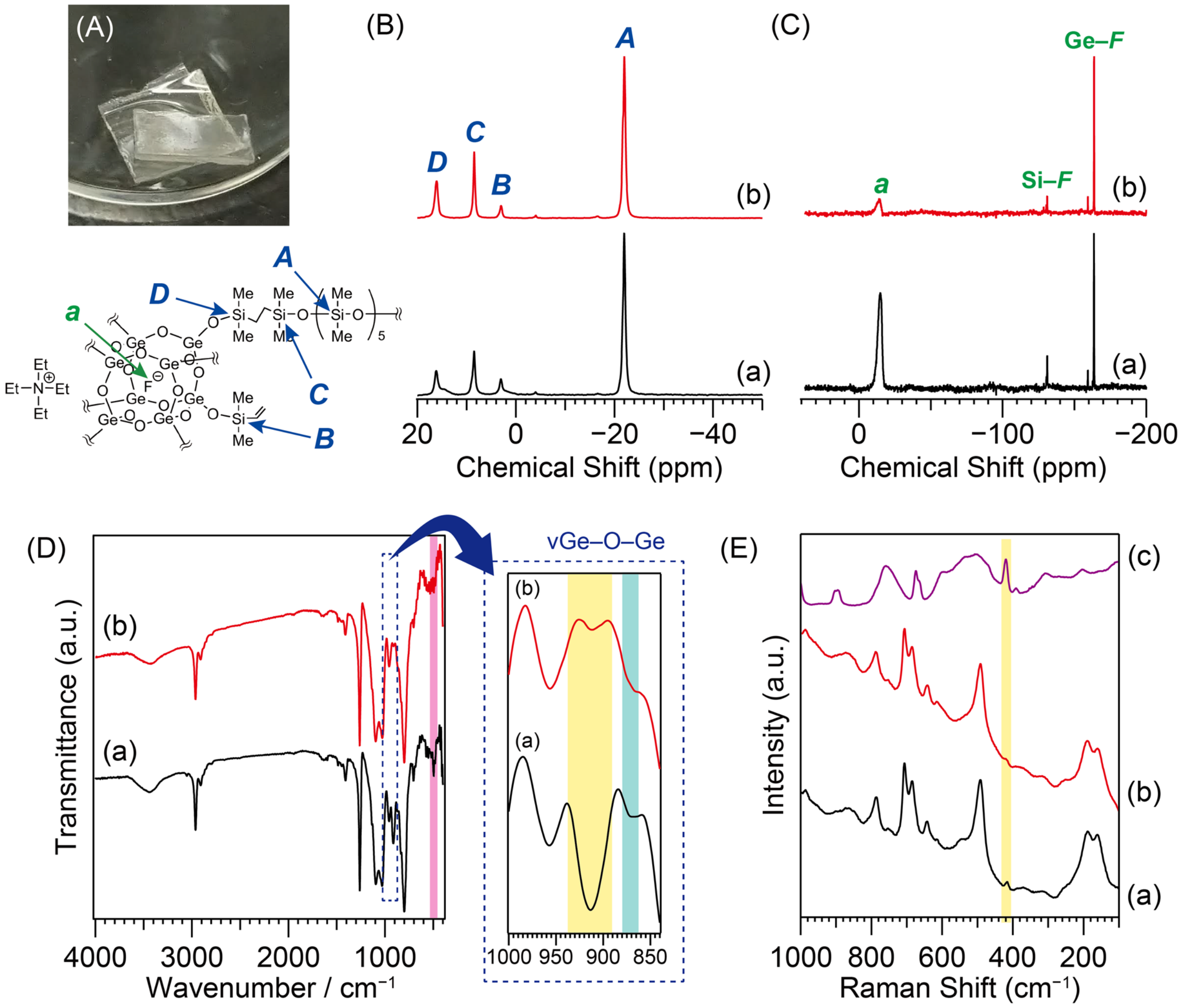

Figure S10. (A) Product appearance of **D6-GeD4R**. (B) ^{29}Si MAS NMR spectra, (C) ^{19}F MAS NMR spectra, (D) FT-IR spectra of **D6-GeD4R** (a) before and (b) after treatment at 60 °C and 80% RH for 1 d. (E) Raman spectra of **D6-GeD4R** (a) before and (b) after treatment at 60 °C and 80% RH for 1 d and (c) **GeD4R-OH**.

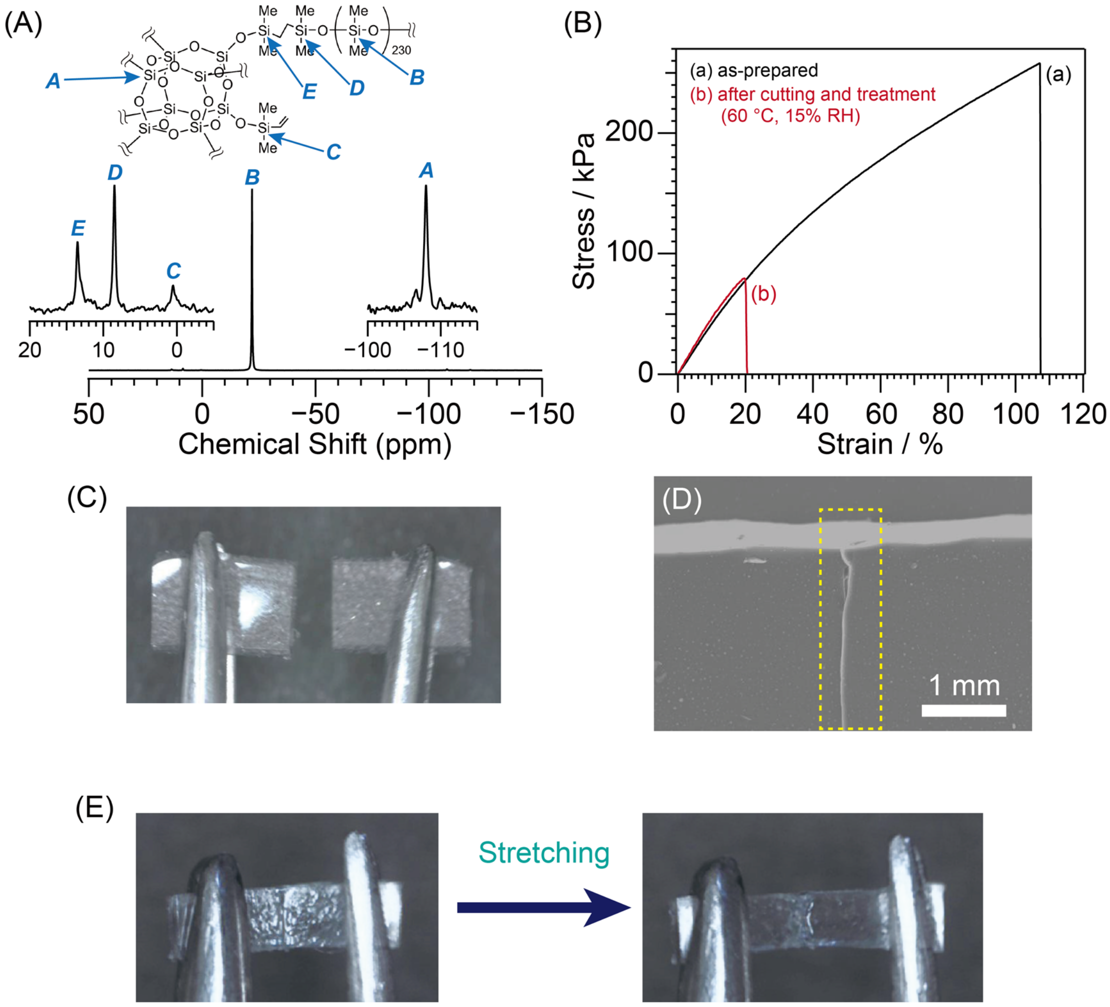

Figure S11. (A) ^{29}Si MAS NMR spectrum of **PDMS-SiD4R**. (B) Stress–strain curves of **PDMS-SiD4R** (a) as-synthesized and (b) after cutting and treatment at 60 °C and 15% RH for 1 d. (C) Product appearance and (D) cross-sectional SEM image of **PDMS-SiD4R** (the yellow dashed frame in the SEM image marks the cut surface) after cutting and treatment at 60 °C and 15% RH for 1 d. (E) Product appearances of **PDMS-SiD4R** after cutting and healing at 40 °C for 1 d with a drop of THF solution of TBAF.

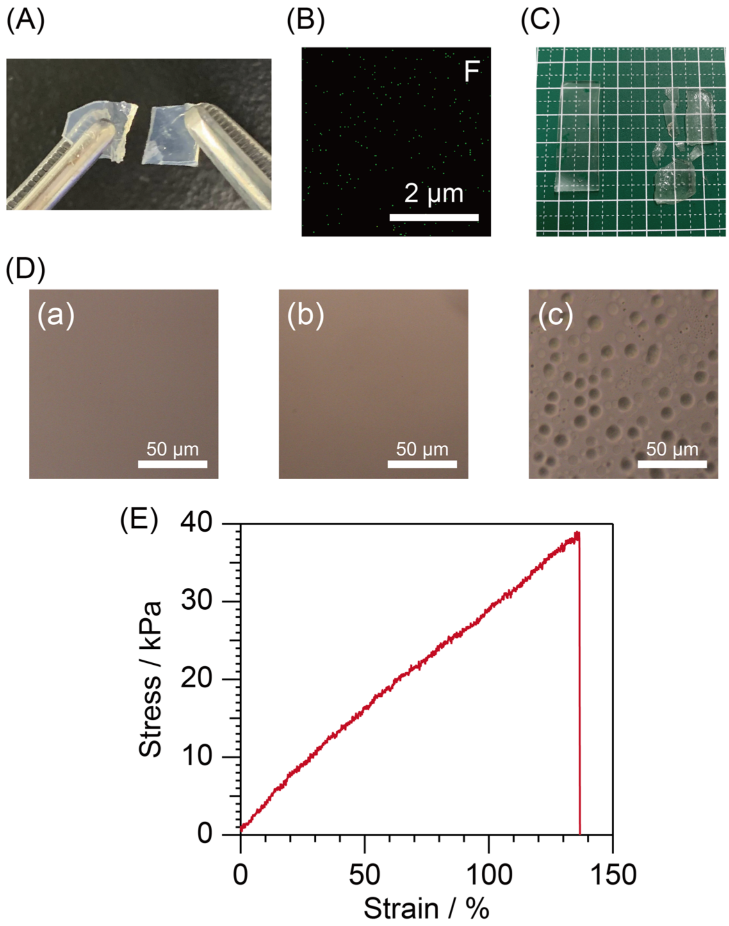

Figure S12. (A) Product appearance of **PDMS-SiD4R-TBAF_1** after cutting and treatment at 60 °C and 15% RH for 1 d. (B) SEM-EDS mapping of fluorine in **PDMS-SiD4R-TBAF_1**. (C) Product appearances of **PDMS-SiD4R-TBAF_2** (left) before and (right) after cutting and treatment at 60 °C and 15% RH for 1 d. (D) Optical microscopy images of (a) **PDMS-SiD4R**, (b) **PDMS-SiD4R-TBAF_1**, and (c) **PDMS-SiD4R-TBAF_2**. (E) Stress–strain curve of **PDMS-SiD4R-TBAF_2**.

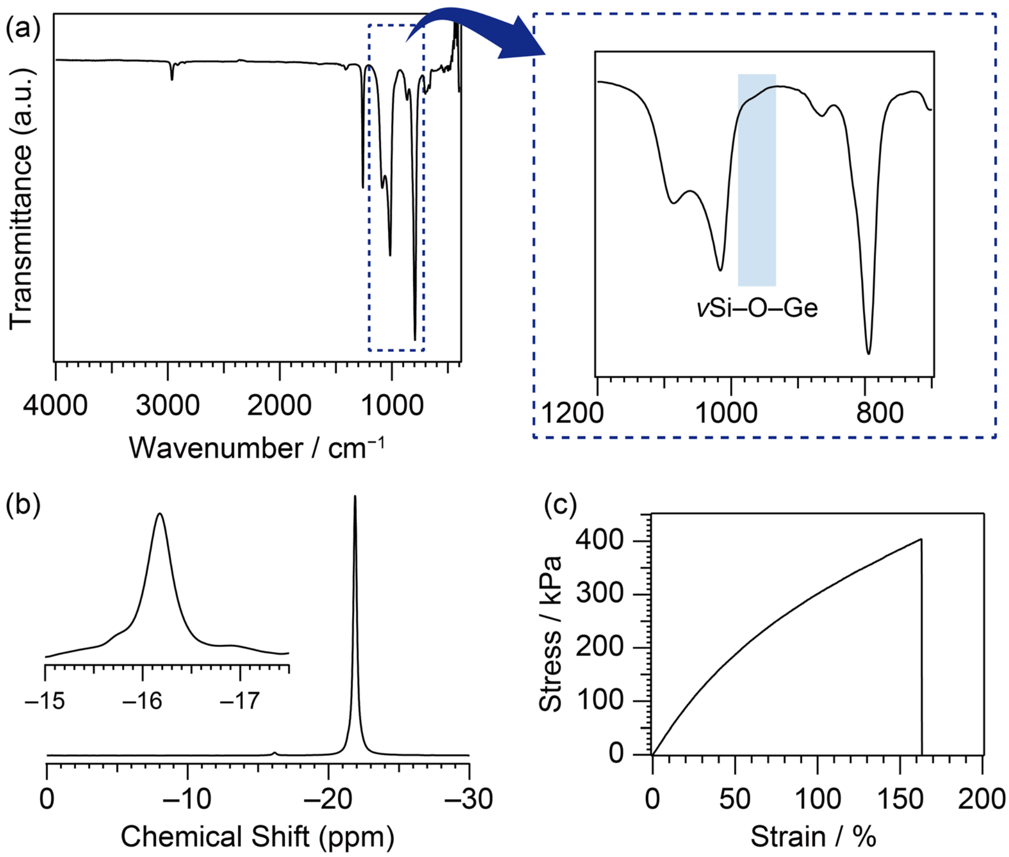

Figure S13. (a) FT-IR spectrum, (b) ^{29}Si MAS NMR spectrum, and (c) stress–strain curve of **PDMS-GeO$_4$**.

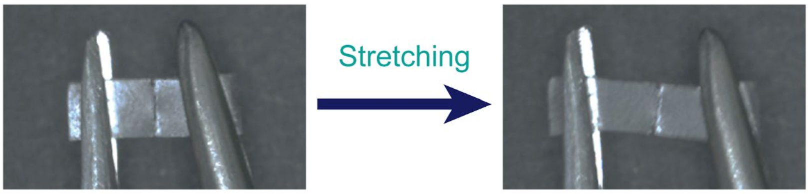

Figure S14. Product appearances of heated **PDMS-GeD4R** (185 °C for 1 d) after cutting and healing at 60 °C and 15% RH for 1 d.

References

[1] ^{29}Si NMR spectrum of hydride terminated polydimethylsiloxane (molecular weight: 580, Sigma Aldrich) showed that the main product was linear hexasiloxane.

[2] a) N. Sato, T. Hayashi, K. Tochigi, H. Wada, A. Shimojima, K. Kuroda, *Chem. Eur. J.* **2019**, *25*, 7860–7865; b) T. Hayashi, N. Sato, H. Wada, A. Shimojima, K. Kuroda, *Dalton Trans.* **2021**, *50*, 8497–8505; c) T. Hayashi, N. Murase, N. Sato, K. Fujino, N. Sugimura, H. Wada, K. Kuroda, A. Shimojima, *Organometallics* **2022**, *41*, 1454–1463.

[3] N. Sato, Y. Kuroda, T. Abe, H. Wada, A. Shimojima, K. Kuroda, *Chem. Commun.* **2015**, *51*, 11034–11037.

[4] S. P. Mukherjee, S. K. Sharma, *J. Am. Ceram. Soc.* **1986**, *69*, 806–810.

[5] C. P. Rosenau, B. J. Jelier, A. D. Gossert, A. Togni, *Angew. Chem. Int. Ed.* **2018**, *57*, 9528–9533.

[6] S. Bourcier, S. Bouchonnet, Y. Hoppilliard, *Int. J. Mass spectrom.* **2001**, *210–211*, 59–69.

[7] a) E. J. Corey, J. O. Albright, *J. Org. Chem.* **1983**, *48*, 2114–2115; b) H. Sun, S. G. DiMagno, *J. Am. Chem. Soc.* **2005**, *127*, 2050–2051.

[8] a) L. A. Villaescusa, P. Lightfoot, R. E. Morris, *Chem. Commun.* **2002**, 2220–2221; b) L. A. Villaescusa, P. S. Wheatley, R. E. Morris, P. Lightfoot, *Dalton Trans.* **2004**, 820–824.

[9] P. Döhlert, J. Pfrommer, S. Enthaler, *Phosphorus, Sulfur Silicon Relat. Elem.* **2016**, *191*, 1189–1193.
